# The Neuronal Overexpression of *Gclc* in *Drosophila melanogaster* Induces Life Extension With Longevity-Associated Transcriptomic Changes in the Thorax

**DOI:** 10.3389/fgene.2019.00149

**Published:** 2019-03-05

**Authors:** Alexey Moskalev, Zulfiya Guvatova, Mikhail Shaposhnikov, Ekaterina Lashmanova, Ekaterina Proshkina, Liubov Koval, Alex Zhavoronkov, George Krasnov, Anna Kudryavtseva

**Affiliations:** ^1^Engelhardt Institute of Molecular Biology, Russian Academy of Sciences, Moscow, Russia; ^2^Institute of Biology of Komi Science Center of Ural Branch of Russian Academy of Sciences, Syktyvkar, Russia; ^3^Department of Biological and Medical Physics, Moscow Institute of Physics and Technology, Dolgoprudny, Russia; ^4^Insilico Medicine, Baltimore, MD, United States

**Keywords:** *Gclc*, glutathione, lifespan, aging, gene expression, thorax, *Drosophila melanogaster*

## Abstract

Some effects of aging in animals are tissue-specific. In *D. melanogaster* neuronal overexpression of Gclc increases lifespan and improves certain physiological parameters associated with health benefits such as locomotor activity, circadian rhythmicity, and stress resistance. Our previous transcriptomic analyses of *Drosophila* heads, primarily composed of neuronal tissue, revealed significant changes in expression levels of genes involved in aging-related signaling pathways (Jak-STAT, MAPK, FOXO, Notch, mTOR, TGF-beta), translation, protein processing in endoplasmic reticulum, proteasomal degradation, glycolysis, oxidative phosphorylation, apoptosis, regulation of circadian rhythms, differentiation of neurons, synaptic plasticity, and transmission. Considering that various tissues age differently and age-related gene expression changes are tissue-specific, we investigated the effects of neuronal *Gclc* overexpression on gene expression levels in the imago thorax, which is primarily composed of muscles. A total of 58 genes were found to be differentially expressed between thoraces of control and *Gclc* overexpressing flies. The *Gclc* level demonstrated associations with expression of genes involved in the circadian rhythmicity, the genes in categories related to the muscle system process and the downregulation of genes involved in proteolysis. Most of the functional categories altered by *Gclc* overexpression related to metabolism including Drug metabolism, Metabolism of xenobiotics by cytochrome P450, Glutathione metabolism, Starch and sucrose metabolism, Citrate cycle (TCA cycle), One carbon pool by folate. Thus, the transcriptomic changes caused by neuron-specific *Gclc* overexpression in the thorax were less pronounced than in the head and affected pathways also differed from previous results. Although these pathways don't belong to the canonical longevity pathways, we suggest that they could participate in the delay of aging of *Gclc* overexpressing flies.

## Introduction

The aging process is determined by the impairment functioning of tissues and systems of an organism. It was shown that aging is accompanied by significant changes in genome-wide gene expression profiles identified for different tissues of vertebrates and invertebrates (Zhan et al., 2007; Kim et al., 2014; White et al., 2015; Cannon et al., 2017; Chen et al., 2018; Zhang et al., 2018). Furthermore, the age-related changes in gene expression are tissue-specific, suggesting that various tissues age differently (Girardot et al., 2006; Zhan et al., 2007; Yang et al., 2015). Recently, a number of studies showed that muscle tissue undergoes sufficient alterations in gene expression patterns during aging in mammals and flies (Kim et al., 2014; Chen et al., 2018; Zhang et al., 2018; Zhou et al., 2018), and revealed the involvement of several tens of messenger RNAs as well as long non-coding, micro, and circular RNAs in this process (Kim et al., 2014; Chen et al., 2018). In particular, aged rats demonstrated decreased expression of components of AMPK, IGF-1, and CASK pathways that can be a key cause of the loss of muscle mass and function (Zhou et al., 2018). The progressive loss of skeletal muscle function and mass, known as sarcopenia, is one of the useful conditions accompanied the aging process in human and underlied the critical mobility reducing and life quality decline (Herndon et al., 2002; Demontis et al., 2013; Larsson et al., 2019). It must be noted, that the aging-related muscle degeneration are also determined by the progressive loss of motoneurons (Larsson et al., 2019). Therefore, interventions that contribute to the slowing down of aging of nervous tissue (including overexpression of pro-longevity genes) can also have a positive effect on the state of muscle tissue and prevent dangerous structural and cellular changes.

According to the recent experimental data, *Drosophila* is a convenient and relevant model for the investigation of muscle degeneration disorders (Demontis et al., 2013; Rai et al., 2014; Kreipke et al., 2017). Despite differences in the composition of mammalian and fly muscle systems, the *Drosophila* model can be used for the identification of key molecular mechanisms of aging-related changes in muscle tissue (Demontis et al., 2013; Rai et al., 2014). Furthermore, the lack of stem cell-based regeneration allows to determine the role of endogenous processes of muscle degeneration (Kreipke et al., 2017). The study of molecular basis of skeletal muscle laminopathies were carried by using of *Drosophila melanogaster LamC* mutants which demonstrated a numbered of muscular pathologic changes and cellular abnormalities. These negative changes were prevented by regulation of AMP-activated protein kinase (AMPK)/TOR/autophagy signaling pathways that highly conserved between flies and humans (Chandran et al., 2018). Thus, the study of mechanistic basis of muscular disorders using *Drosophila* allows to reveal potential targets for therapeutic interventions.

The *Gclc* is a target gene of pro-longevity SKN-1/Nrf2 transcription factor, activated in response to oxidative stress, and implicated in the lifespan extension of flies, worms, and mice (Orr et al., 2005; Luchak et al., 2007; Sykiotis et al., 2011; Mockett and Nobles, 2013; Blackwell et al., 2015). It codes for the catalytic subunit of glutamate-cysteine ligase, that participates in glutathione formation (Orr et al., 2005). Age-dependent downregulation of glutamate-cysteine ligase is associated with a decline in glutathione content, which is involved in multiple cellular functions including antioxidant defense and detoxification of reactive intermediates (Liu et al., 2004; Orr et al., 2005). Previously, it was demonstrated that the neuron- and muscle-specific overexpression of *Gclc* is associated with increased lifespan in flies, but not the ubiquitous overexpression of *Gclc* (Orr et al., 2005; Luchak et al., 2007; Mockett and Nobles, 2013). In addition, *Gclc* overexpression in the nervous system postpones age-associated decline in circadian rhythmicity and a variety of physiological parameters like locomotor activity and resistance to the oxidative, osmotic, and proteotoxic stresses (Moskalev et al., 2016). *Gclc* overexpression also affects transcription patterns of genes that govern diverse cellular processes such as Jak-STAT, MAPK, FOXO, Notch, mTOR, TGF-beta signaling pathways, translation, protein processing in endoplasmic reticulum, proteasomal degradation, glycolysis, oxidative phosphorylation, apoptosis, regulation of circadian rhythms, differentiation of neurons, synaptic plasticity, and transmission in *Drosophila* heads (Moskalev et al., 2016).

We supposed, that neuron-specific *Gclc* overexpression may ameliorate the decline of muscular function and delay the aging of muscle tissue (Moskalev et al., 2016). For the first time, we studied the effect of neuronal overactivation of the pro-longevity gene on age-related transcriptome changes in the muscular system. We used total RNA sequencing and revealed that *Gclc* overexpression in the head also affected the key signaling pathways implicated in the aging process in the thorax, which is composed primarily of muscle tissue. We identified 58 differentially expressed genes. Among the functional categories [Gene Ontology (GO) terms or Kyoto Encyclopedia of Genes and Genomes (KEGG) pathways] the genes consistently overrepresented were associated with Drug metabolism, Metabolism of xenobiotics by cytochrome P450, Glutathione metabolism, Starch and sucrose metabolism, Neuroactive ligand-receptor interaction, One carbon pool by folate, vesicle, and Cdc73/Paf1 complex. While apparent, the transcriptomic changes in the thorax were less pronounced than in the head.

## Materials and methods

### *Drosophila melanogaster* Lines

Flies were separated by sex and maintained in different vials on a standard sugar-yeast medium at constant temperature (25°C) and humidity (60%) in a 12:12 h light-dark cycle in Binder KBF720-ICH (Binder, Germany) climate chamber. The following strains were used: UAS-*Gclc* (provided by Dr. William C. Orr, Southern Methodist University) and *Appl*-GAL4 (#32040, Bloomington *Drosophila* Stock Center). They were backcrossed with *w*^1118^ (#3605, Bloomington *Drosophila* Stock Center, USA) 6–8 times to equilibrate the genetic background. To activate the *Gclc* constitutive overexpression in the nervous system, the UAS-*Gclc* was crossed with *Appl*-GAL4 driver. Newly enclosed progenies were maintained for one, four, and 6 weeks before dissection. The same aged flies from the parental UAS-*Gclc* line were used as a contro control.

### Total RNA Extraction and Qualification

The experimental design was the same as in our previous work (Moskalev et al., 2016). Thoraxes in this study and heads in the previous study were collected from the same flies. In brief, we used males and females' thorax of *UAS-Gclc* flies as control and thoraxes of flies with *Gclc* overexpression. A comparison of flies with different chronological ages, namely, 1-, 4-, and 6- weeks was conducted. The ages were designated as “young,” “matured,” and “old,” respectively. The flies were collected, frozen and kept at −80°C to prevent RNA degradation. For each experimental variant, 40 flies were selected. Three biological replicates of the experiment were performed. The males and females were analyzed separately to reveal any sex-dependent differences in gene expression caused by neuronal Gclc expression. The RNA used for next-generation sequencing was extracted from 30 flies (10 flies per each replicate) using QIAzol Lysis Reagent (Qiagen, Netherlands) and precipitated with isopropanol. Qubit®2.0 Fluorometer (Invitrogen, USA) and NanoDrop® ND-1000 spectrophotometer (NanoDrop Technologies Inc., USA) were used to estimate the RNA concentration and purity. The A260/A280 ratio in the samples was around 1.8–2.0. RNA was additionally purified using DNase I (Promega, USA). The RNA integrity was estimated with Bioanalyzer Agilent 2100 (Agilent Technologies, USA).

### Library Preparation and Sequencing Procedure

mRNA was isolated from the total RNA with NEBNext Oligo d(T)25 beads. The fragmentation was performed at 94°C with addition of First Strand Synthesis Reaction Buffer and Random Primer mix (2**×**). The double-stranded (ds) cDNA was synthetized by using ProtoScript II Reverse Transcriptase and Second Strand Synthesis Enzyme Mix. The blunt ends on the ds cDNA were created with the end-repair reaction. The adapters were ligated using the specific RNA Adapter Indexes supplied in the kit. The PCR reaction was performed to selectively enrich adaptor ligated DNA fragments and create a large amount of DNA, which was then purified using Ampure XP beads. The quantity and quality of libraries were assessed on Rotor-Gene 6000 PCR System (Qiagen, USA) using the qPCR method and on Agilent 2100 Bioanalyzer using a High Sensitivity DNA chip. The final length of the libraries averaged 260 bp. The libraries were normalized to 4 nM, pooled together in equal volumes, and sequenced with 2 **×** 50 bp paired-end reads on the NextSeq 500 System (Illumina, USA). The obtained data were stored in FASTQ format. The minimum amount of reads for each sample was 20 million.

### NGS Data Processing

We used PPLine pipeline to perform quality control, read mapping, and counting (Krasnov et al., 2015). In the details, reads were analyzed using FastQC and trimmed with trimmomatic (Bolger et al., 2014). To evaluate the efficacy of the isolation of polyA fraction and the absence of bacterial contamination, 100.000 randomly selected reads from each sample were mapped to *D.Melanogaster* rRNA genes and bacterial genomes (all strains that had been submitted to NCBI Genome through 2015), accordingly. Typically, rRNA ratio was 0.3–1.7% (except for 2 samples with 4%). The trimmed reads were mapped to the *D.Melanogaster* genome (assembly BDGP6, Ensembl release 90) using splice-aware STAR aligner (Dobin et al., 2013). About 90–95% reads were uniquely mapped. Since the samples had demonstrated different RNA integrity numbers (RIN), in order to compare gene expression levels, we take into account a 3′-tail bias as described in Sigurgeirsson et al. (2014) with some modifications. Without a 3′-bias adjustment, one should expect false-positive overexpression of short transcripts (for example, genes of mitochondrial proteins or ribosomal proteins) and under-expression of genes with long transcripts and encoded proteins (for example, multi-domain transmembrane proteins interacting with extracellular matrix).

First, we analyzed read coverage across transcript length using a modified version of geneBody_coverage.py script, part of RSeQC package (Wang et al., 2012). In the samples with lower RIN numbers and more degraded RNA, we observed rapid falling down of read coverage level after 750–1,000 bp from the 3′-tail. The coverage in the region of 0–500 bp from the 3′-tail was almost preserved for all samples.

Then, we quantified transcripts using RSEM (Li and Dewey, 2011) and identified the most abundantly expressed alternative transcript for each gene. The other transcripts were discarded. Then, the transcripts were truncated to the length of 500 bp from the 3′-end and a new GTF file was generated. Finally, using this gene model, we quantified reads using Counts from Subread package (Li and Dewey, 2011). This procedure allowed us to significantly reduce the dependence of the observed expression level fold changes (FC) on the transcript lengths, between the samples with higher and lower RINs.

In order to more effectively eliminate the factor of the 3′-bias, we proceeded the following way. First, we excluded genes with low expression level by introducing a threshold: CPM > 2 for at least 3 samples. About 9,000 genes passed the threshold. Next, we split all the genes into 10 bins, depending on the average gene transcript length, and within each bin we normalized read counts using the TMM (trimmed mean of M-values) method from edgeR (Robinson et al., 2010). Finally, the bins were merged.

A similar procedure was performed to eliminate the dependency of the expression level FC on the “absolute” gene expression level (in terms of read counts per million, CPM). This bias may also occur when comparing samples with different RINs and initial RNA concentration.

Finally, the adjusted read pseudo-counts were analyzed by edgeR using exact test and the quasi-likelihood ratio *F*-test (Robinson et al., 2010). Gene Ontology, KEGG, and Reactome enrichment was performed using topGO and clusterProfiler Bioconductor packages (Yu et al., 2012). For this purpose, we used Fisher exact test and lists of top 50, 100, 200, 500, and 1,000 up- or down-regulated genes that passed *p* < 0.05 threshold. Fisher test was applied for each of these top lists, and then the results were merged. As a background, we took only genes that passed the pre-filtering by the expression level. KEGG pathways visualization was performed using modification of pathview package (Luo and Brouwer, 2013) as described earlier (Moskalev et al., 2016).

### Reverse Transcription and qPCR Analysis

The total RNA isolated for the RNA-Seq library preparation was used for qPCR analysis. First-strand cDNA was synthesized using 0.5 μg of total RNA, random primers and Mint reverse transcriptase (Evrogen, Russian Federation). Real-time quantitative PCR reactions were performed in triplicate in the presence of the EvaGreenTM dye (Biotium Inc., USA) and gene specific primers (Table 1) on the ABI 7500 Fast Real-Time PCR System (Applied Biosystems, USA). The cycling parameters were 95°C for 10 min, followed by 40 cycles of 95°C for 15 s, 60°C for 60 s, and 72°C for 30 s. *act5c* and *tub84B* were used as reference genes. The relative expression ratios were calculated using the 2–ΔΔCt method as previously described (Dmitriev et al., 2016). The Wilcoxon and Mann-Whitney tests were applied for assessment of differences in mRNA expression. *P* ≤ 0.05 were considered statistically significant.

**Table 1:** Nucleotide sequences of primers used for qPCR.

**Gene**	**Forward primer (5′-3′)**	**Reverse primer (5′-3′)**	**Product length (in bp)**
*Mlp60A*	cgggtagaaccttcgtgaga	gtgcgaggcatgcaagattt	73
*Act88F*	ctcggctcggacagtgatag	gctataccgctgccgatgaa	144
*Arc1*	catcatcgagcacaacaacc	ctactcctcgtgctgctcct	169
*act5c*	gagcgcggttactctttcac	acttctccaacgaggagctg	133
*tub84B*	aacctgaaccgtctgattgg	ggtcaccagagggaagtgaa	134

## Results

**Figure 1:**
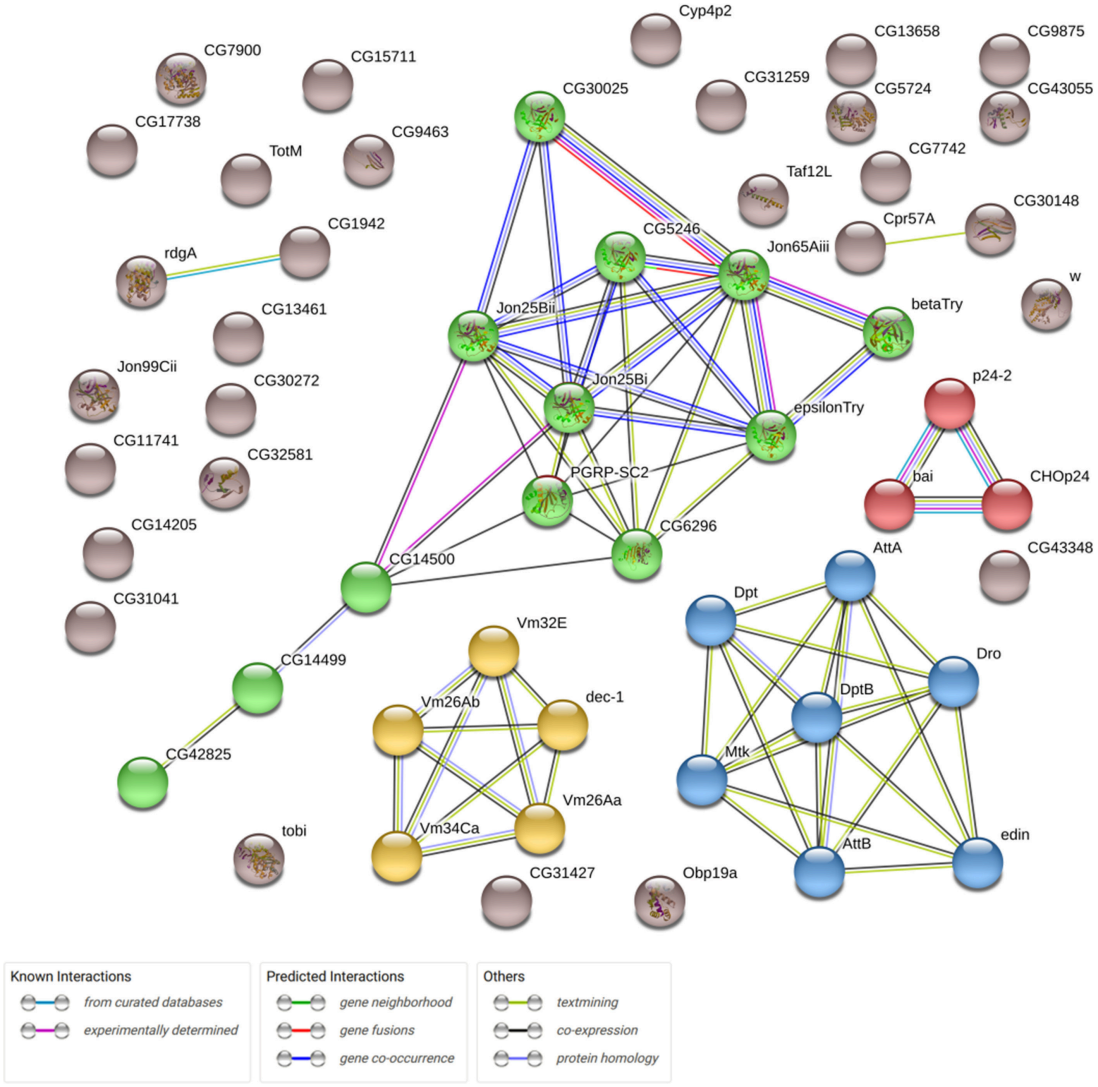
Network demonstrating associations (protein interactions, co-expression, etc.) between 58 genes, expression of which is altered in transgenic flies (overexpressing *Gclc*; STRING database). Clusters formed by genes are marked different colors.

In this study, we analyzed the effects of *Gclc* overexpression in the neuronal tissues of *D. melanogaster* on gene expression profiles in the thorax. We derived RNA-Seq expression profiles for 9,000 genes (after eliminating the genes with low expression). *Drosophila* aging was followed by the differential expression of 2626 genes (*p* < 0.05). 1488 of 2626 passed FDR < 0.05 threshold. The expression of 667 genes (*p* < 0.05) was associated with the activation of *Gclc* in all groups: young/mature/old or males/females (48 of 667 have FDR < 0.05). 58 of 667 genes demonstrated 2-fold or higher expression level changes (Supplementary Table 1). Using the STRING (Search Tool for Genesis/Proteins database) we received some information on the interactions of proteins encoded by these genes and showed that the most enriched pathway is the defense response to Gram-positive bacterium (FDR = 0.0019; Figure 1). As can be seen, the genes included in the proteolysis GO category (highlighted in green) also form a cluster. It is known that there is an increase in proteolysis of myofibril proteins during muscular dystrophy (Costelli et al., 2005; Combaret et al., 2009). According to the results of the DE analysis, overexpression of *Gclc* in thorax repressed genes involved in the proteolysis process.

We also verified the expression levels of some muscle-related genes using the qPCR method (Supplementary Table 2 and Supplementary Figure 1), the results showed a similar change in expression of *Mlp60A, Act88F*, and *Arc1* genes compared with the DE analysis.

**Figure 2:**
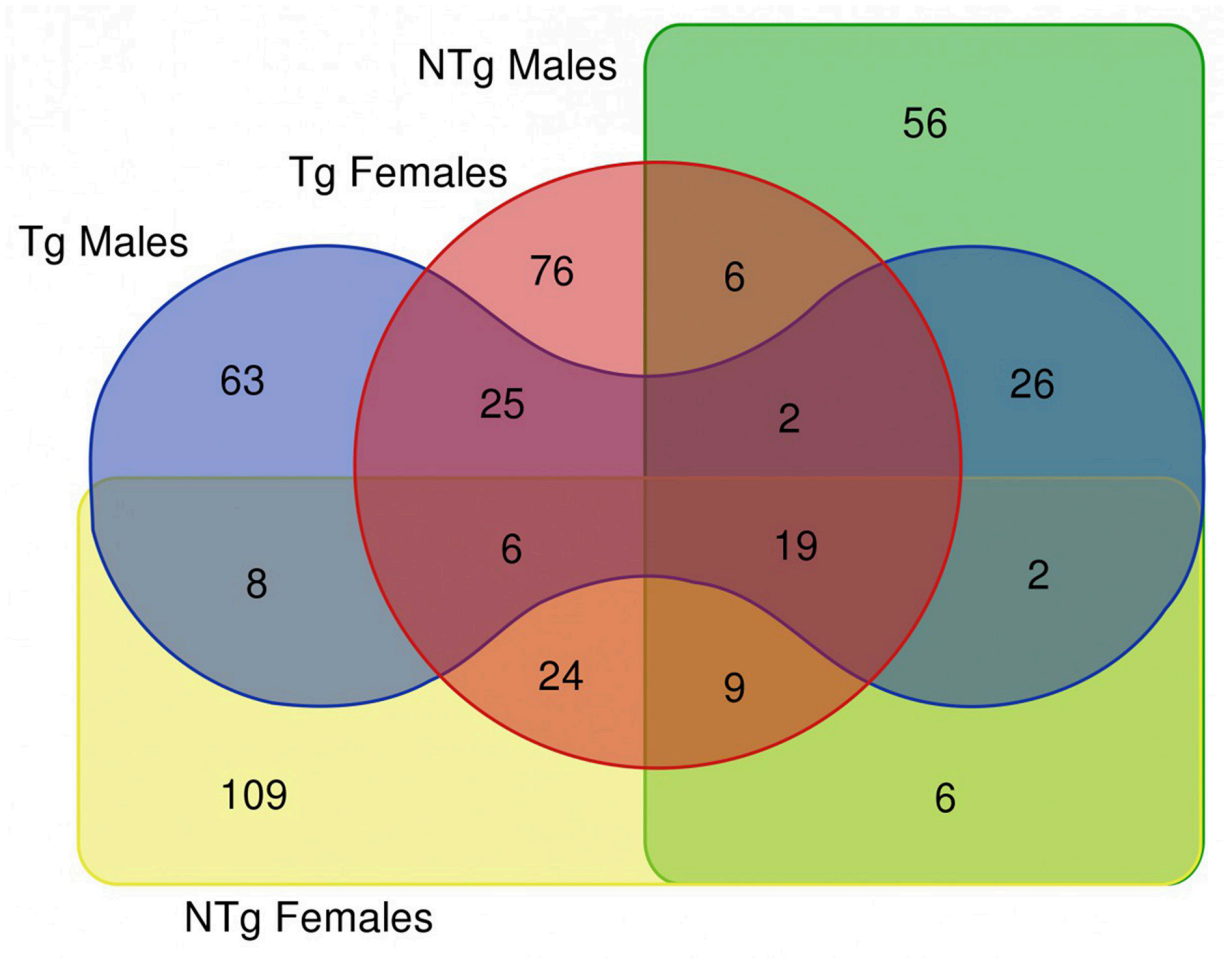
Venn diagram illustrating the results of differential gene expression analysis (old vs. young flies) in various groups of samples. Tg-transgenic, Gclc-overexpressing; NTg-non-transgenic. In Venn diagram, we included differentially expressed genes that passed thresholds: fold change > 4 and *p* < 0.05.

The survival analysis of the examined flies demonstrated that the increase of median and maximum lifespan of transgenic *Drosophila* is more pronounced in females than in males (Moskalev et al., 2016). In this study, we compared the resulting DE genes list (old vs. young comparisons for males and female's groups) as depicted in the Venn diagram of Figure 2, which resulted in 19 common DE genes shared by all 4 lists, 25 DE genes shared by Tg males and Tg females. There are only 6 genes common in NTg males and NTg females lists of DE genes. These genes are presented in Supplementary Table 3.

KEGG pathway enrichment analysis suggested that the activation of transgenic *Gclc* is followed with the differential expression of genes involved in a variety of cellular processes including Drug metabolism, Metabolism of xenobiotics by cytochrome P450, Glutathione metabolism, Starch and sucrose metabolism, Neuroactive ligand-receptor interaction, One carbon pool by folate, vesicle, and Cdc73/Paf1 complex (Fisher test *p* < 0.05).

**Figure 3:**
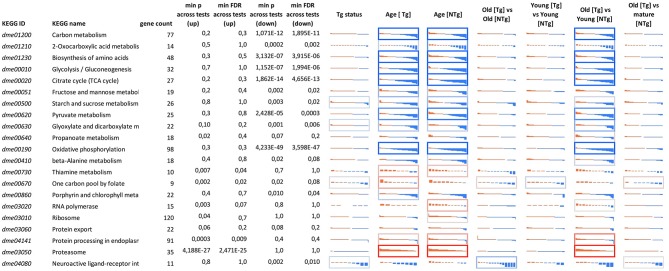
Differential expression profiles for genes participating in cellular pathways for different 641 genotypes (*UAS-Gclc* flies designated as Tg, *Appl-GAL4* > *UAS-Gclc* flies—as NTg) and ages (old, mature, young). Logarithmic Fold Change (LogFC) (vertical axis) is ranged from −2 to +2, i.e., from four-fold decrease (blue) to 4-fold increase (red). Cell borders demonstrate the statistical significance of gene set enrichment analysis (Fisher test *p*-value): blue—enriched with down—regulated genes; and red—enriched with overexpressed ones min *p*-value across tests (up)—minimal enrichment *p*-value (Fisher exact test) across tests, only for up-regulated genes; min *p*-value across tests (down)—minimal enrichment *p*-value (Fisher exact test) across tests, only for down-regulated genes. DE genes that passed threshold LogCPM > 3 were presented.

Considering age-associated expression changes, we found predominant up-regulation of genes participating “One carbon pool by folate” pathway and a slight down-regulation of genes involved in Porphyrin metabolism and Neuroactive ligand-receptor interaction in *Appl-GAL4* > *UAS-Gclc* flies (Supplementary Figure 2). For *UAS-Gclc* flies, we found aging-associated up-regulation of Genetic Information Processing, including the RNA polymerase, Ribosome, Protein export, and Protein processing in endoplasmic reticulum, whereas in the experimental group these changes were not observed (Figure 3 and Supplementary Figure 2).

Using the Reactome pathway database, we also identified several pathways enriched with genes, the expression of which is altered by *Gclc* overexpression. Among them: response to CaM pathway, DAG and IP3 signaling, Amino acid synthesis and interconversion (transamination), Peroxisomal lipid metabolism, Pyrimidine, and Purine catabolism (Supplementary Table 4).

## Discussion

Overall, our data demonstrate that the neuronal *Gclc* overexpression resulted in less pronounced effects on the gene expression, the key signaling pathways involved in the aging process in the thorax than in the head. For example, the differential expression (DE) analysis revealed that only 58 genes (|LogFC|>1, *p* < 0.05) were differentially expressed in the thorax, while in the head, the neuronal *Gclc* overexpression altered the expression level of 188 genes. In heads, the *Gclc* overexpression influenced the following pathways: oxidative phosphorylation, ribosome biogenesis, Wnt, mTOR/PI3K, FOXO pathways, and enhanced cAMP signaling (Moskalev et al., 2016). In the thorax, no statistically significant effects of the neuronal *Gclc* overexpression were observed.

**Figure 4:**
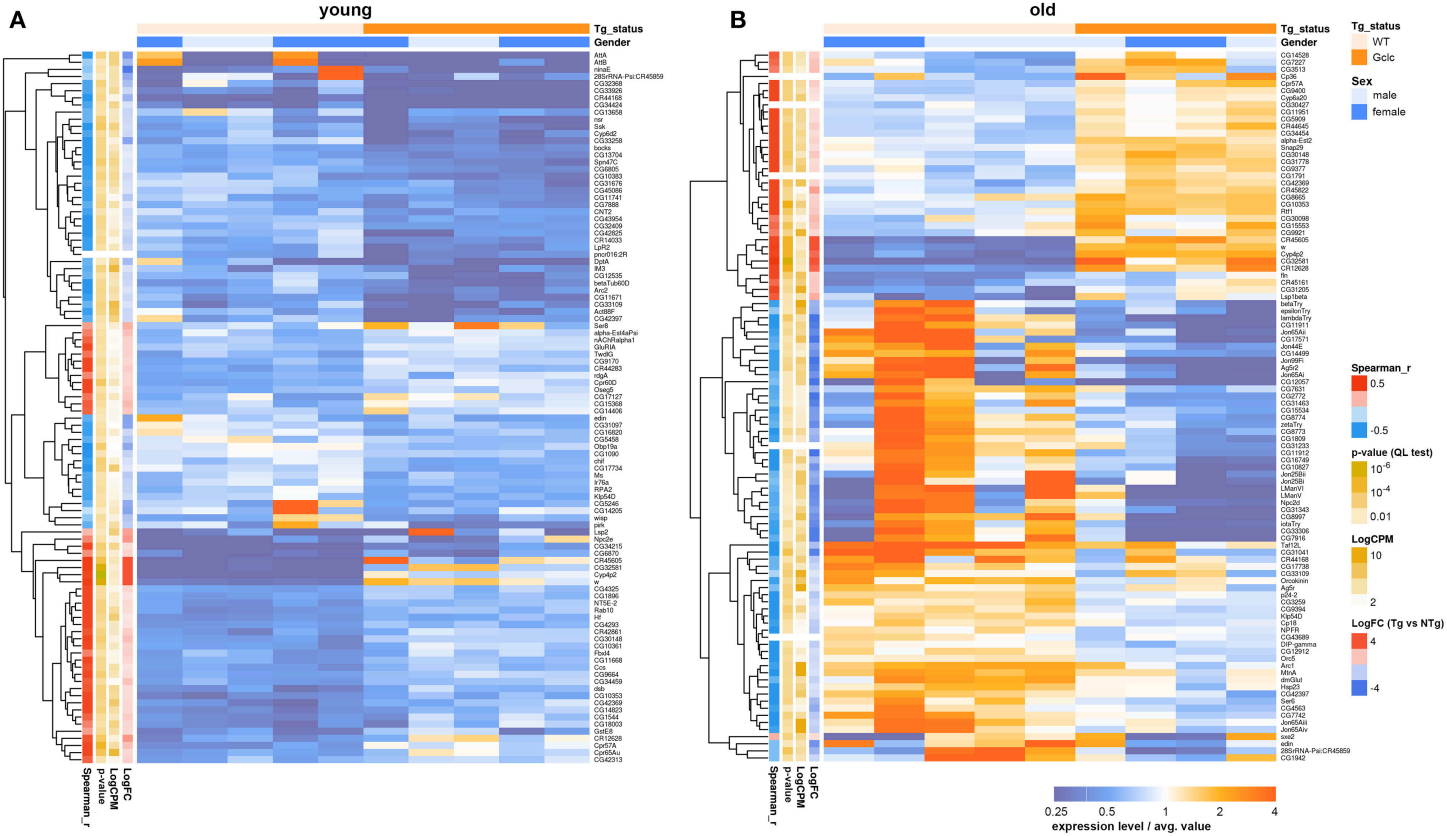
Heatmap illustrating differential gene expression profiles between transgenic (Tg) and non-transgenic (NTg) organisms: young **(A)** and old **(B)** flies. Heatmap colors indicate gene expression level in each sample, normalized to the average gene expression level across all the samples. Orange-above the average, blue-below the average value. LogFC-binary logarithm of gene expression level fold change between Tg and NTg organisms; *p*-value—edgeR's quasi-likelihood *F*-test *p*-value; LogCPM—binary logarithm of read counts per million; Spearman_r—Spearman rank correlation coefficient between Tg/NTg status and gene expression is presented.

Only two genes: *cytochrome P450 4p2 (Cyp4p2)* and *white (w)*, were differentially expressed in both the head and in the thorax. The *Cyp4p2* gene encodes a member of the cytochrome P450 superfamily of enzymes. The *w* gene, famous for its role as the importer of eye pigments precursor, also plays a role in memory and cGMP transport (Evans et al., 2008; Sitaraman et al., 2008). The null mutation of this gene in *D. melanogaster* was associated with decreased lifespan, stress-resistance, and locomotor activity (Ferreiro et al., 2017). Using the GML model “~Gender + Genotype,” we indicated that the effect of *Gclc* expression on the transcriptional profile of the thorax is increased with age (Figure 4). In contrast, comparing the transcriptional profile of the *Drosophila* heads with *Gclc* overexpression demonstrated that the differences in gene expression are more pronounced in young flies than in old flies (Radyuk et al., 2012). There is data that the aging process in muscles is more dramatic than in the nervous system (Herndon et al., 2002). These results demonstrate a different contribution of *Gclc* overexpression in tissue-specific aging. According to our results, the DE genes in the thoraxes associated with aging are less than the age-related DE genes of the heads. On the other hand, the Zhan et al. in their work showed that the muscle had the largest number of age-related genes than the heads and other tissues of *Drosophila* (Zhan et al., 2007). The authors suggest that a characteristic of aging in muscle is the modulation of expression of genes involved in proteasomal and mitochondrial functions. Our data showed that the most of age-dependent up-regulated genes in thoraxes were associated with Protein processing in endoplasmic reticulum, Proteasome, RNA polymerase pathways, while down-regulated genes are related to metabolism and biosynthesis, such as Oxidative phosphorylation, Citrate cycle (TCA cycle), Biosynthesis of amino acids, Glycolysis/Gluconeogenesis, Glyoxylate and dicarboxylate metabolism, 2-Oxocarboxylic acid metabolism, Pyruvate metabolism, and carbohydrate metabolism. The age-related disorder of oxidative phosphorylation is well-known for different *Drosophila* body parts and other model organisms (Kim et al., 2005; Lesnefsky and Hoppel, 2006; Cannon et al., 2017). Cannon et al. also showed that carbohydrate metabolism was down-regulated in aged fly hearts, consisting of cardiac muscle (Cannon et al., 2017). This is consistent with our data. In addition, the age-dependent decrease in the expression level of citric acid cycle genes was observed in the heads of *D. melanogaster* and *C. elegans* (Mccarroll et al., 2004).

It is known that aging is accompanied by the decline of muscle mass and strength (Demontis et al., 2013). This phenomenon is called sarcopenia and is associated with impaired physical performance, cognitive function, and mortality (Gonzalez-Freire et al., 2018; Landi and Calvani, 2018; Van Ancum et al., 2018). One of the major factors contributing to the development of sarcopenia may be the decline in antioxidant protection by glutathione (Mosoni et al., 2004; Sullivan-Gunn and Lewandowski, 2013). Previously, we demonstrated that neurospecific overexpression of *Gclc* delay aging-associated decline of the spontaneous locomotor activity of *D. melanogaster* males and females, compared to controls (Moskalev et al., 2016). The slowing down of aging-associated decline of muscular function may be linked with 16–29% increase in glutathione content that was detected by Orr et al. in the *Drosophila* headless bodies (thoraces plus abdomens) due to neurospecific *Gclc* overexpression (Orr et al., 2005).

**Figure 5:**
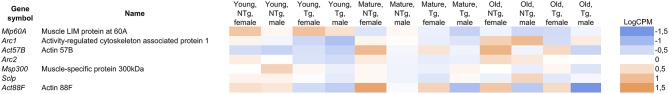
The differentially expressed genes in the categories related to the muscle system process. LogCPM (Counts per Million) is ranged from −1.5 to +1.5. Shades of different colors show the range of expression values (blue-down-regulated; orange-up-regulated). *UAS-Gclc* flies designated as Tg, *Appl-GAL4* > *UAS-Gclc* flies—as NTg.

While the imago thorax is primarily composed of muscle tissues, several DE genes are muscle-specific. We observed a 1.3–2-fold decrease in the level of muscle-specific protein genes in *D. melanogaster* thorax with *Gclc* overexpression (Figure 5). Using microarray analysis, Montana et al. demonstrated that the expression of muscle-specific proteins (*Act88F, Act57B, Mlp60A, Msp300*) and the homolog of the neuronal activity-regulated protein, ARC is up-regulated in *Drosophila* hypercontraction muscle mutants (Montana and Littleton, 2006). The authors concluded that the hypercontraction of muscles resulted in activation of actin cytoskeleton re-modulation mechanisms (Montana and Littleton, 2006). Among the differentially expressed genes in categories related to the muscle system process, we also observed that the *Sclp* gene was slightly decreased. Kuelzer et al. showed that *Sclp* plays an important role in programmed cell death of muscle (Kuelzer et al., 1999). In comparisons of old transgenic flies with the appropriate control group, we found a 1.8-fold increase in the expression of the genes (*hbs* and *tum*) involved in the fusion between myoblasts for the formation of syncytial myofibrils (Guerin and Kramer, 2009; Shelton et al., 2009). Sohn et al. showed that *nephrin*, the vertebrate homolog of the *hbs*, affects the development of the muscles and absence of *nephrin* results incompletely fused myotubes (Sohn et al., 2009). The *tum* gene also is conserved in *C. elegans, Drosophila*, humans, and involved in somatic muscle patterning (Guerin and Kramer, 2009). In the same comparison, the *fln* gene encoding muscle-specific protein, which is necessary for adult flies, was up-regulated more than two times (Henkin et al., 2004; Schnorrer et al., 2010). In old transgenic flies, we found an increase in the expression of the *rotated abdomen (rt*) gene encoding the POMT1 protein. POMT1 has a highly conserved homolog in nematodes and humans (Lyalin et al., 2006). It has been shown that the mutation of POMT1 results in muscular dystrophy and associated neural defects in the mammals (Akasaka-Manya et al., 2004; Haines et al., 2007). These results shed light on the mechanisms underlying delay of age-related locomotor activity decline in flies with *Gclc* overexpression (Moskalev et al., 2016).

The analysis of KEGG pathways demonstrated that the One carbon pool by folate KEGG pathway was up-regulated during aging of *D. melanogaster* with *Gclc* overexpression compared with age-related changes in the control group (Supplementary Figure 2). These results correspond with the current paradigms. One-carbon metabolism is a crucial metabolic pathway that is connected with multiple physiological processes like *de novo* nucleotide synthesis and DNA methylation, and also provides a variety of metabolic intermediates which react either with pro-oxidants or promote antioxidant defense (Cavallaro et al., 2010; Vijaya Lakshmi et al., 2013).

Majumdar et al. demonstrated that folic acid and vitamin B12 can reduce the level of oxidative stress induced by the administration of arsenic trioxide. The authors consider that the prevention of mitochondrial dysfunction occurs due to the activation of antioxidant defense enzymes such as superoxide dismutase and catalase, and level of antioxidant glutathione (Majumdar et al., 2009). Vitamin B12 and folic acid play an important role in metabolism of homocysteine (Sahin et al., 2003). Elderly people with lower levels of folate and vitamin B12 have high homocysteine concentration, which is seen as a predictor of potential health problems such as Alzheimer's disease, cardiovascular disease, and loss of cognitive function (Koehler et al., 1997; Selhub, 2002). The up-regulation of this pathway was also observed by us in *Drosophila* flies exposed to different stress conditions like starvation, cold shock, or irradiation (Moskalev et al., 2015).

In our prior work, we demonstrated that the neuronal overexpression of *Gclc* gene resulted in a more pronounced increase of the median and maximum lifespan in *Drosophila* females than in males (Moskalev et al., 2016). Female longevity is observed in different taxa, but not well understood. In the present study, we found that overexpression of *Gclc* gene causes slight upregulation of *Sod2* gene (in females), which participates Longevity regulating KEGG pathway. SOD acting in conjunction with glutathione provide a protective mechanism against destructive superoxide radicals and were shown to be involved in lifespan regulation of various model organisms including *Drosophila* species (Sohal et al., 2002; Tower, 2015). The *Sod2* gene activity was associated with increased lifespan in *Saccharomyces cerevisiae* (Fabrizio et al., 2003; Unlu and Koc, 2007; Laschober et al., 2010), *Drosophila melanogaster* (Kirby et al., 2002; Curtis et al., 2007), and *Mus musculus* (Treiber et al., 2011). Male groups overexpressing *Gclc* exhibited no significant increases in *Sod2* expression in comparison with their respective controls. We also showed that among the 25 DE genes found only in the *Gclc*-overexpressing female's comparison group (Supplementary Table 3), most of the genes are participants of the carbohydrate metabolism. Disruption of the carbohydrate metabolism and high-sugar intake are implicated in altering glucose homeostasis, influencing in a negative way the development, fertility, and lifespan in yeasts, worms, fruit flies, mammals (Wagner et al., 2015; Ravichandran et al., 2017; Alcantar-Fernandez et al., 2018). It is noteworthy that there are 3 Maltase genes (*Mal-A3 Mal-A4, Mal-A7*), the differential expression of which were found only in transgenic females. A recent study conducted by Inomata and co-workers identified that the maltase enzymes are responsible for dietary carbohydrate changes and could increase the capacity of the response associated with environmental changes (Inomata et al., 2019).

The sleep process is a physiological behavior observed in almost all organisms. The sleep process is controlled by two processes: sleep homeostasis and circadian rhythm (Borbely and Achermann, 1999). It is known that these two processes can work independently, but if they directly influence each other is still questionable (Deboer, 2018). In our prior studies, analysis demonstrated that the neuronal *Gclc* gene overexpression declined age-dependent alterations in circadian rhythmicity (Moskalev et al., 2016). These effects were accompanied by increased mRNA levels of a number of genes, which products are involved in regulation of circadian rhythms. It is known that the expression of these genes is regulated by the mechanism of a negative feedback loop. In brief, this loop can be described as follows: The CLOCK/BMAL1 heterodimers bind to the *Per* and *Cry* promoters to initiate their transcription. The protein products of *Per* and *Cry* genes form a complex in the cytoplasm with other proteins, such as CK1ε, and translocate back to the nucleus, where they inhibit the CLOCK/BMAL1 complex and repress transcription of their own and other genes (Yu et al., 2006; Lowrey and Takahashi, 2011). In contrast, in this study, the Reactome pathway analysis revealed several down-regulated pathways involved in circadian rhythms, such as Degradation of PER, Degradation of CRY, and Circadian Clock pathway. We found that *heimdall (hll)* gene encoding the long-chain fatty acid-CoA ligase, was significantly overexpressed (threshold of 4-fold) in *Appl-GAL4* > *UAS-Gclc* flies. Recently, Thimgan et al. demonstrated that the expression of *hll* alters lipid metabolism (Thimgan et al., 2015). Interestingly, it was also demonstrated that *hll* can modulate sleep homeostasis (Thimgan et al., 2015). Lipid metabolism and sleep are related to each other according to Taheri et al. (2004) and Nedeltcheva et al. (2009). A number of genes associated with lipid metabolism can modulate lifespan of model organisms (Bolger et al., 2014). Thus, *Gclc* overexpression had some effects on both processes that control sleep.

*Gclc* overexpression in the *D. melanogaster* thorax up-regulated the *rdgA* gene. Inoue et al. revealed that the *rdgA* gene codes for an ATP-dependent Diacylglycerol kinase (DAGK) that converts DAG to phosphatidic acid (Inoue et al., 1989). According to the Reactome pathway analysis, the elements of phosphatidic acid biosynthetic process were significantly up-regulated in the thorax of flies with *Gclc* overexpression. Acting as signaling molecules, members of the DAG kinase family can take part in various biological processes such as synaptic transmission, photoreceptor transduction, and acquired and innate immunity (Merida et al., 2008). However, Lin et al. observed that the knockdown of *rdgA* gene in *D. melanogaster* and its ortholog *dgk-5* in *C. elegans* both resulted in increased lifespan of model organisms. They assumed that the effects were associated with reduced TOR signaling (Lin et al., 2014).

In *D. melanogaster* with *Gclc* overexpression, we observed 1.5–4-fold increase in expression of some genes involved in eggshell chorion formation (*Cp18, Vm26Aa, Vm34Ca*). Several genome-wide transcriptome studies showed that the expression levels of genes, which participates in eggshell chorion formation, decline with age (Pletcher et al., 2002; Carlson et al., 2015).

Using the GLM model “~Age + Gender + Genotype,” we compared the expression profiles of old *Appl-GAL4* > *UAS-Gclc* flies with control groups of different ages. The old flies with *Gclc* overexpression consistently demonstrated expression profiles more similar to control mature flies than control flies of the same age. We found that the *Dsh* gene involved in canonical and non-canonical Wnt signaling pathways was up-regulated in old *Appl-GAL4* > *UAS-Gclc* flies. Disheveled (Dsh) acts directly downstream of frizzled receptors and serves as an intermediary between frizzled and glycogen synthase kinase-3β. Dsh also participates in the planar cell polarity (PCP) pathway and the Wnt/Ca^2+^ signaling pathway. The PCP pathway regulates the proper orientation of wing hairs and thoracic bristles in *Drosophila* (Kramer, 2016).

Thus, neuronal *Gclc* overexpression in *Drosophila* induces tissue-specific transcriptomic changes in the thorax that may be associated with anti-aging effects such as delay of age-related alterations in locomotor activity and circadian rhythmicity. However, for more substantiated conclusions about the causative link between overexpression of *Gclc* in neurons, tissue-specific transcriptomic changes in muscles and anti-aging effects on the organismal level, more experimental evidences may be required.

## Data Availability

The sequencing data are available through the NCBI Sequence Read Archive (project ID PRJNA474212).

## Author Contributions

AM, MS, GK, ZG, EL, and AZ wrote the manuscript text. EP, MS, LK, ZG, and EL carried out the experiments. GK and ZG carried out the bioinformatic analysis. AM and AK supervised the bioinformatic research and text of the manuscript. All authors read and approved the final manuscript.

### Conflict of Interest Statement

AZ was employed by company Insilico Medicine. The remaining authors declare that the research was conducted in the absence of any commercial or financial relationships that could be construed as a potential conflict of interest.

## Abbreviations

bp: base pairs
DAG: Diacylglycerol
DAGK: Diacylglycerol kinase
ds cDNA: double-stranded cDNA
*Gclc*: Glutamate-cysteine ligase catalytic subunit
GO: Gene Ontology
DE: differentially expressed
FC: fold changes
FDR: false discovery rate
KEGG: Kyoto Encyclopedia of Genes and Genomes
PCP: planar cell polarity pathway.
