# Supplementary material for: The Neuronal Overexpression of Gclc in Drosophila melanogaster Induces Life Extension With Longevity-Associated Transcriptomic Changes in the Thorax
Source: Front Genet. 2019 Mar 5;10:149. doi: 10.3389/fgene.2019.00149 (PMC6411687; doi:10.3389/fgene.2019.00149)
Supplement: Supplementary file 5 [file Data_Sheet_1.pdf]

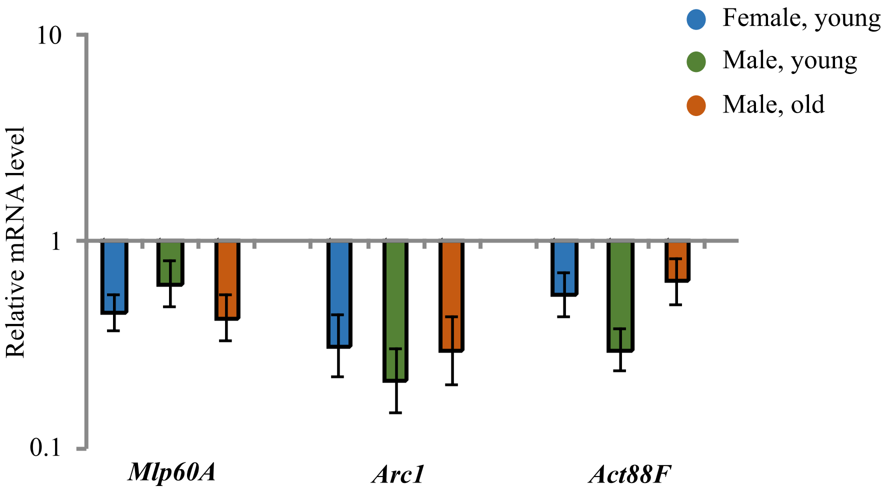

Supplementary Figure 1. Relative mRNA level of *Mlp60A, Act88F* and *Arc1* genes in *Gclc* overexpressing flies compared to non-transgenetic controls. QPCR data
