# Supplementary material for: The Neuronal Overexpression of Gclc in Drosophila melanogaster Induces Life Extension With Longevity-Associated Transcriptomic Changes in the Thorax
Source: Front Genet. 2019 Mar 5;10:149. doi: 10.3389/fgene.2019.00149 (PMC6411687; doi:10.3389/fgene.2019.00149)
Supplement: Supplementary file 6 [file Data_Sheet_2.pdf]

Supplementary Figure 2. The pathway changes during aging of flies with/
without overexpression of *Gclc* gene.

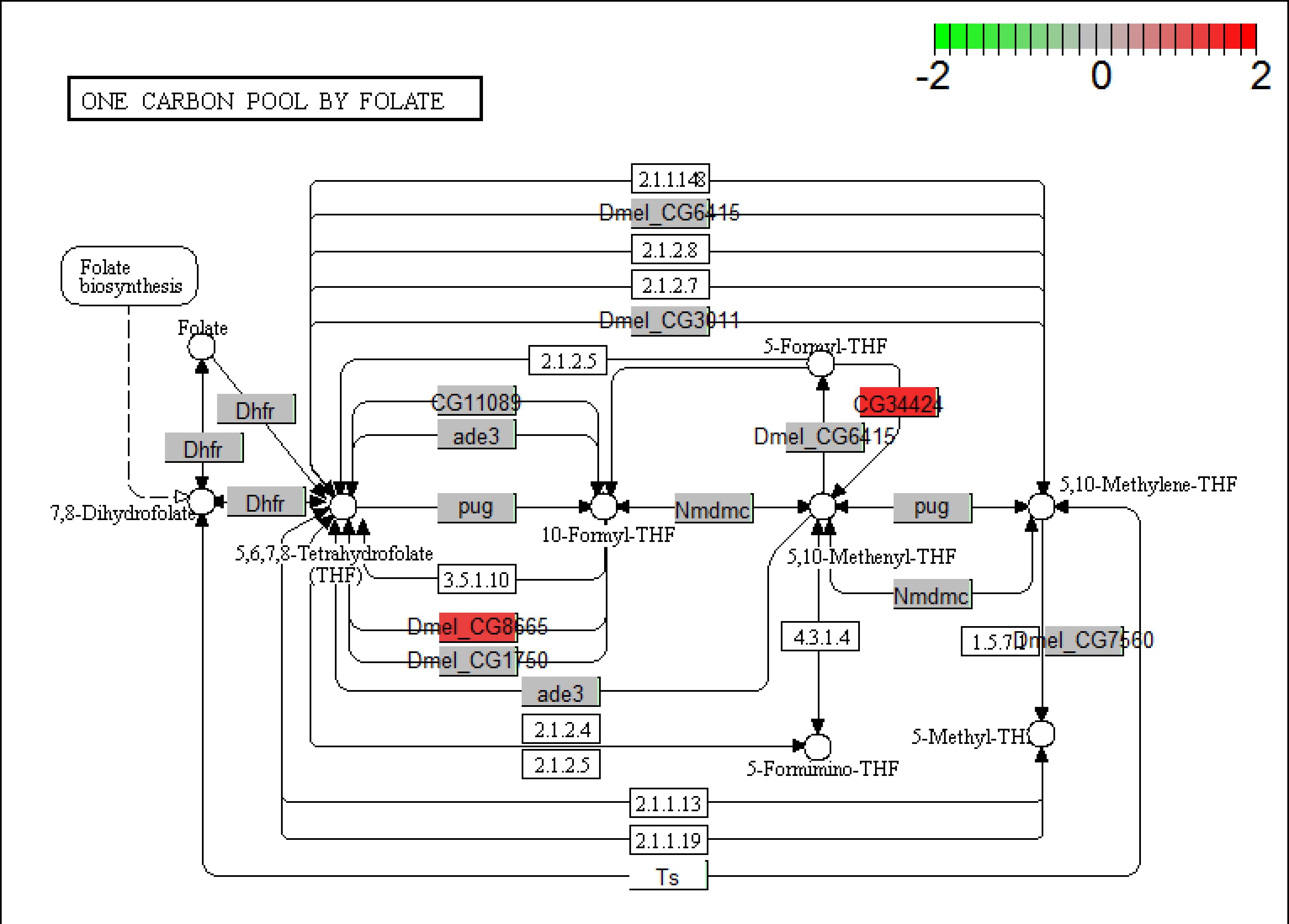

Data on KEGG graph
Rendered by Pathview

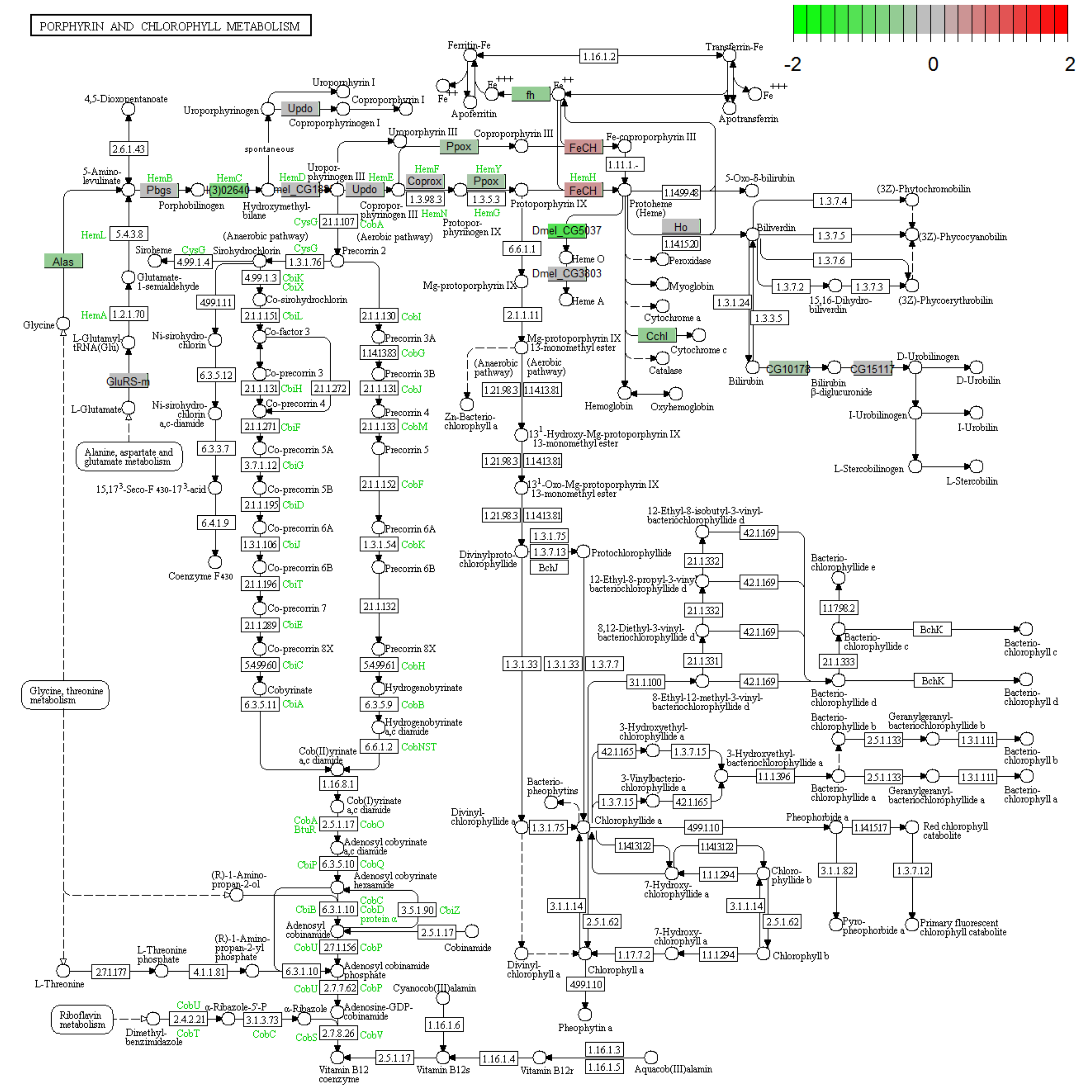

Data on KEGG graph
Rendered by Pathview

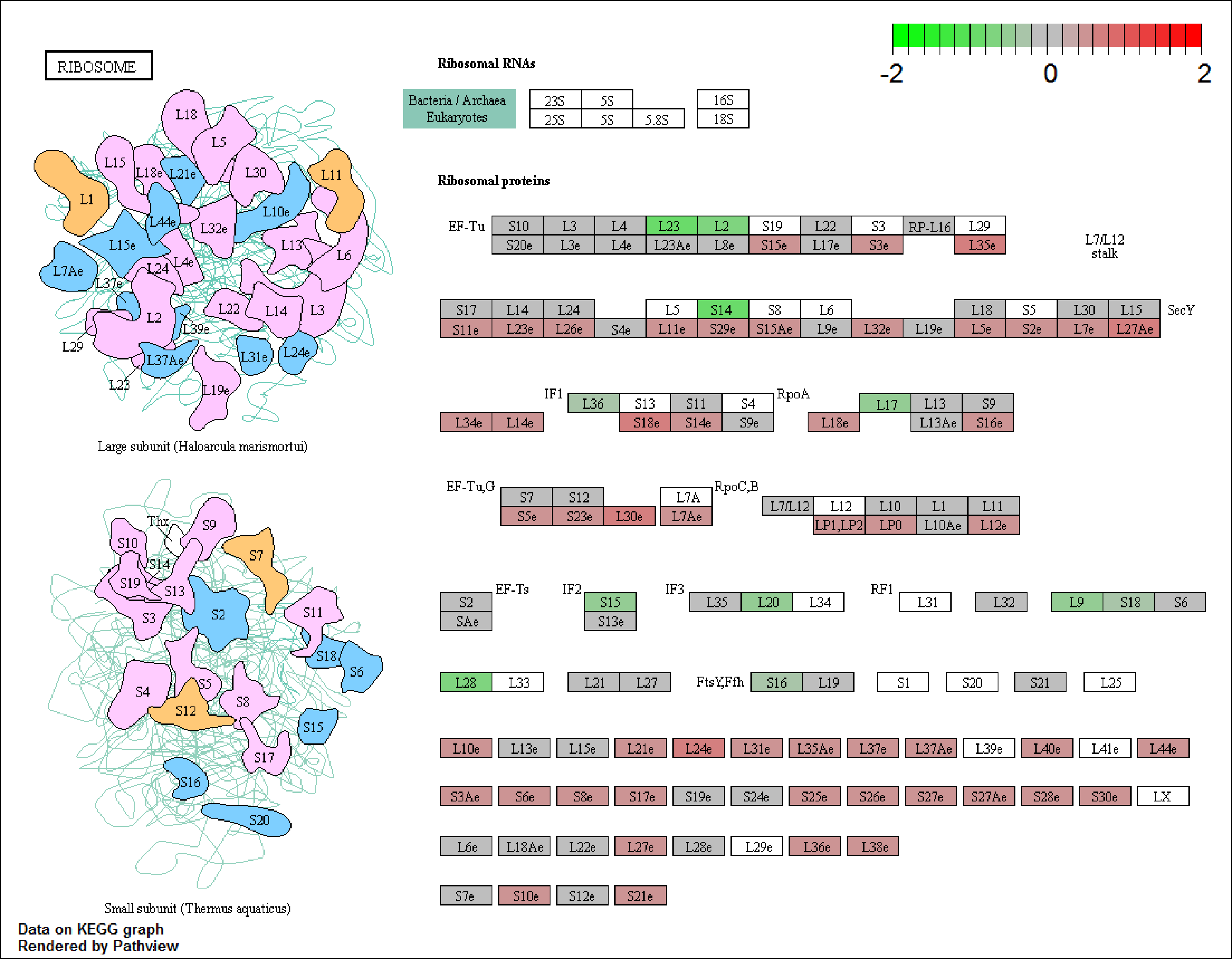
RIBOSOME
-2
0
2
Ribosomal RNAs
Bacteria / Archaea
Eukaryotes
23S 5S 16S
25S 5S 5.8S 18S
Ribosomal proteins
EF-Tu
S10 L3 L4 L23 L2 S19 L22 S3 RP-L16 L29
S20e L3e L4e L23Ae L8e S15e L17e S3e L35e
L7/L12 stalk
S17 L14 L24 L5 S14 S8 L6 L18 S5 L30 L15
S11e L23e L26e S4e L11e S29e S15Ae L9e L32e L19e L5e S2e L7e L27Ae
SecY
IF1
L36 S13 S11 S4
L34e L14e S18e S14e S9e
RpoA
L17 L13 S9
L18e L13Ae S16e
EF-Tu,G
S7 S12
S5e S23e L30e
L7A
L7Ae
RpoC,B
L7/L12 L12 L10 L1 L11
LP1,LP2 LP0 L10Ae L12e
S2
SAe
EF-Ts
IF2
S15
S13e
IF3
L35 L20 L34
RF1
L31
L32
L9 S18 S6
L28 L33
L21 L27
FtsY,Ffh
S16 L19
S1
S20
S21
L25
L10e L13e L15e L21e L24e L31e L35Ae L37e L37Ae L39e L40e L41e L44e
S3Ae S6e S8e S17e S19e S24e S25e S26e S27e S27Ae S28e S30e LX
L6e L18Ae L22e L27e L28e L29e L36e L38e
S7e S10e S12e S21e
L18 L5 L15 L18e L21e L30 L11 L1 L10e L44e L32e L15e L13 L6 L7Ae L24 L4e L37e L2 L22 L14 L3 L39e L29 L37Ae L31e L24e L23 L19e
Large subunit (Haloarcula marismortui)
Thx S9 S10 S14 S7 S19 S13 S2 S3 S11 S18 S6 S4 S5 S8 S12 S15 S17 S16 S20
Small subunit (Thermus aquaticus)
Data on KEGG graph
Rendered by Pathview

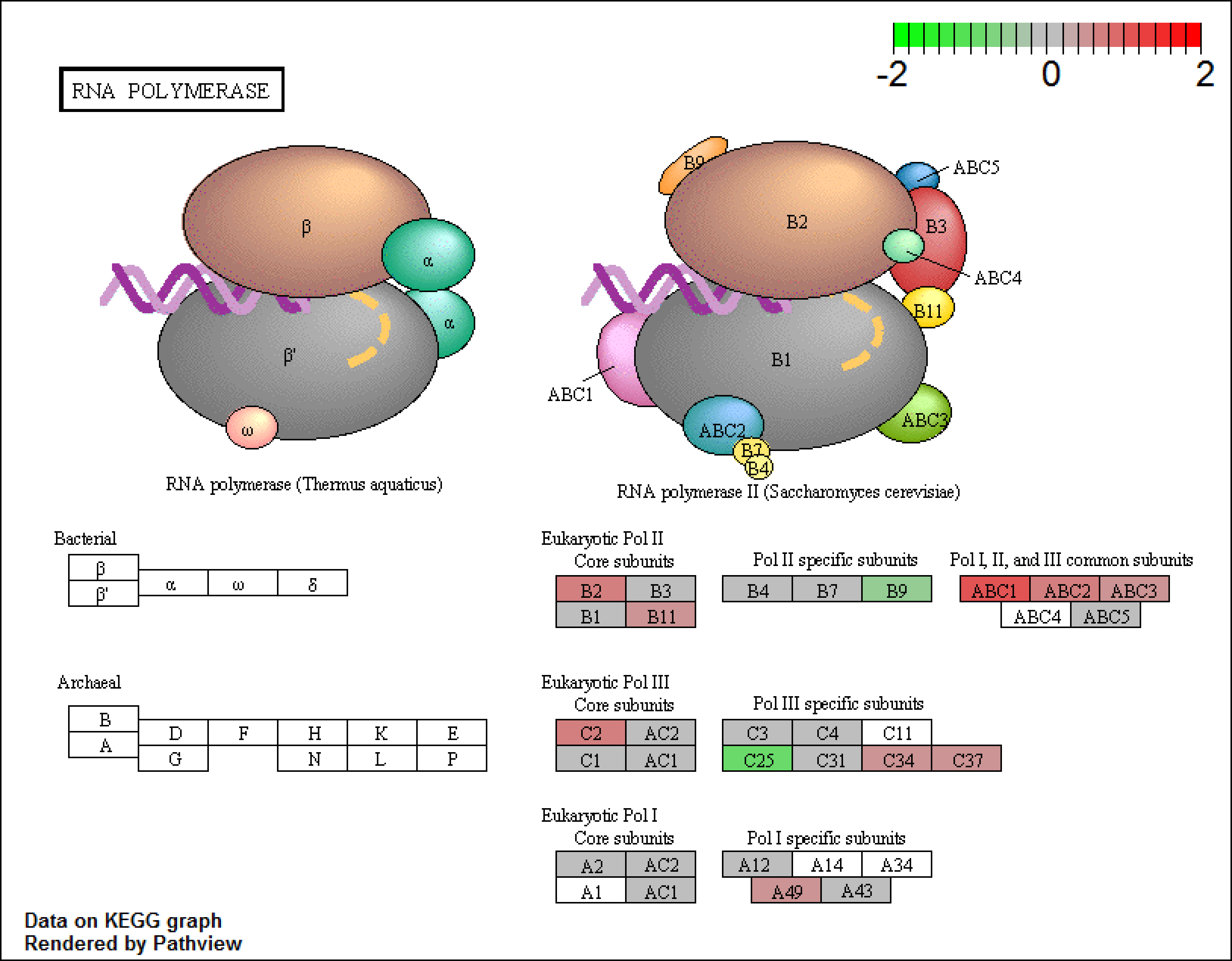
-2
0
2
RNA POLYMERASE
β
α
α
β'
ω
RNA polymerase (Thermus aquaticus)
B9
ABC5
B2
B3
ABC4
B11
B1
ABC1
ABC2
ABC3
B7
B4
RNA polymerase II (Saccharomyces cerevisiae)
Bacterial
β
β'
α
ω
δ
Eukaryotic Pol II
Core subunits
B2
B3
B1
B11
Pol II specific subunits
B4
B7
B9
Pol I, II, and III common subunits
ABC1
ABC2
ABC3
ABC4
ABC5
Archaeal
B
A
D
F
H
K
E
G
N
L
P
Eukaryotic Pol III
Core subunits
C2
AC2
C1
AC1
Pol III specific subunits
C3
C4
C11
C25
C31
C34
C37
Eukaryotic Pol I
Core subunits
A2
AC2
A1
AC1
Pol I specific subunits
A12
A14
A34
A49
A43
Data on KEGG graph
Rendered by Pathview

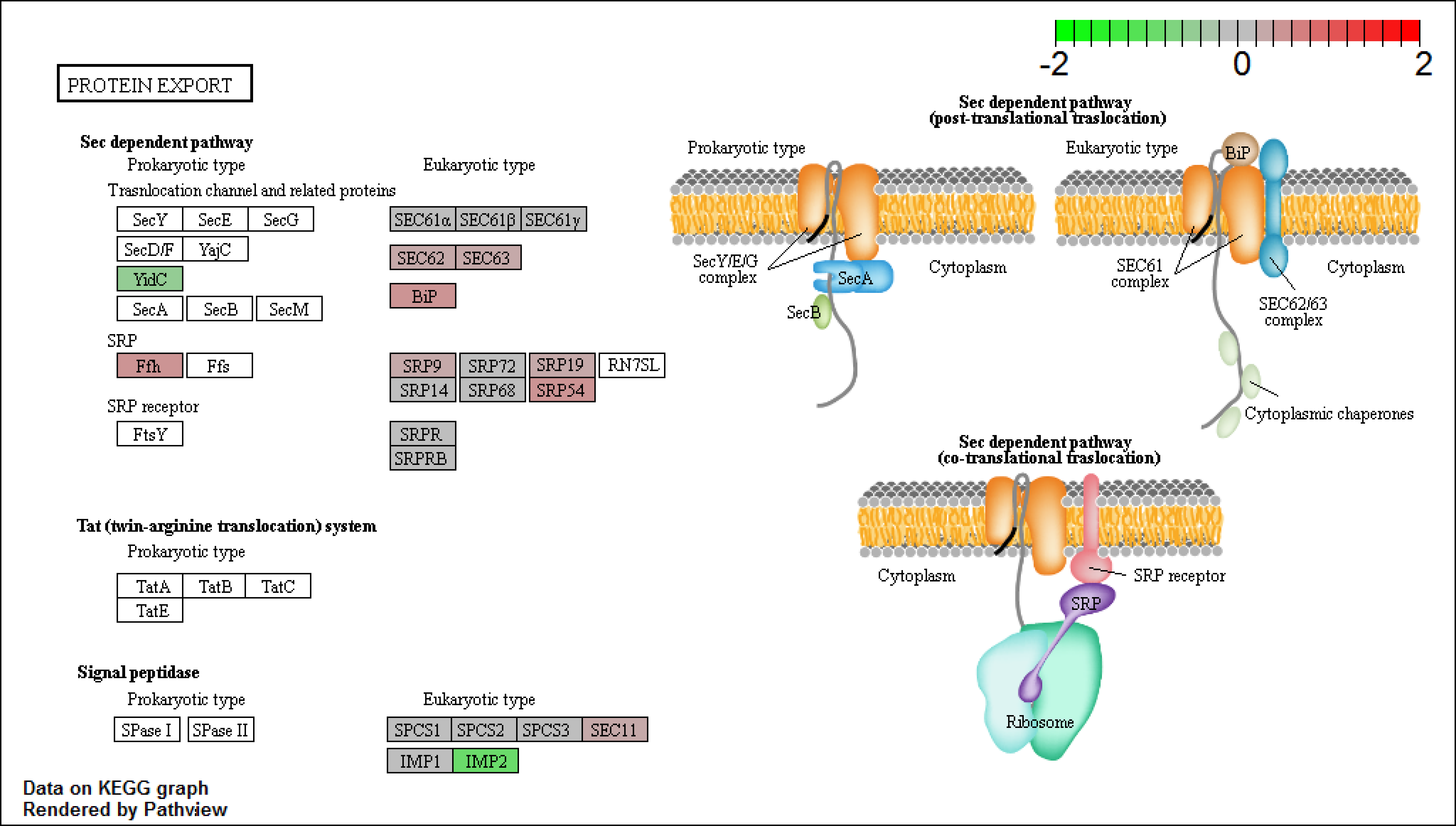
-2
0
2
PROTEIN EXPORT
Sec dependent pathway
Prokaryotic type
Eukaryotic type
Trasnlocation channel and related proteins
SecY
SecE
SecG
SEC61α
SEC61β
SEC61γ
SecD/F
YajC
SEC62
SEC63
YidC
BiP
SecA
SecB
SecM
SRP
Ffh
Ffs
SRP9
SRP72
SRP19
RN7SL
SRP14
SRP68
SRP54
SRP receptor
FtsY
SRPR
SRPRB
Tat (twin-arginine translocation) system
Prokaryotic type
TatA
TatB
TatC
TatE
Signal peptidase
Prokaryotic type
Eukaryotic type
SPase I
SPase II
SPCS1
SPCS2
SPCS3
SEC11
IMP1
IMP2
Sec dependent pathway
(post-translational traslocation)
Prokaryotic type
Eukaryotic type
BiP
SecY/E/G
complex
SecA
SecB
Cytoplasm
SEC61
complex
SEC62/63
complex
Cytoplasm
Cytoplasmic chaperones
Sec dependent pathway
(co-translational traslocation)
Cytoplasm
SRP receptor
SRP
Ribosome
Data on KEGG graph
Rendered by Pathview

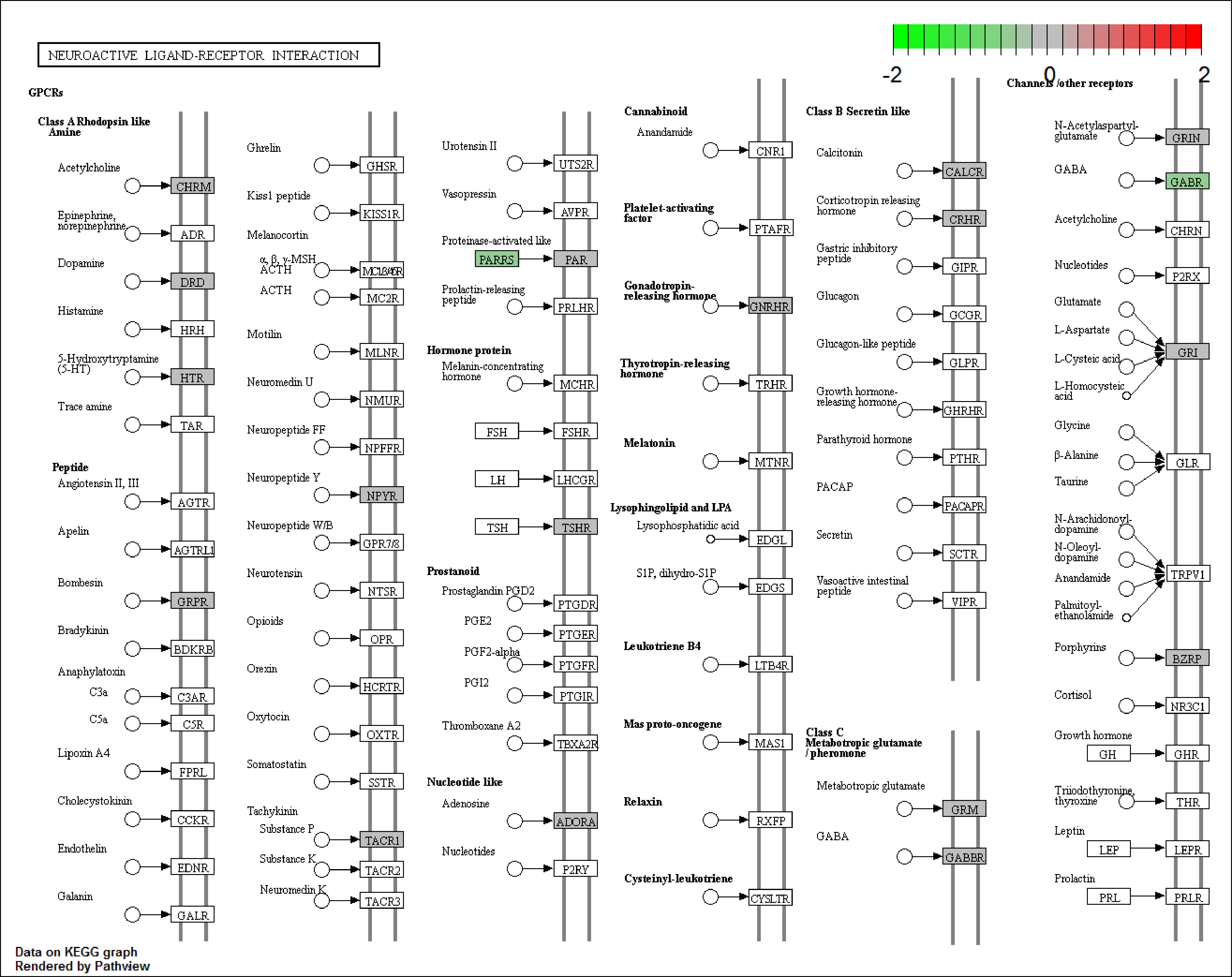
NEUROACTIVE LIGAND-RECEPTOR INTERACTION
-2
0
2
GPCRs
Class A Rhodopsin like
Amine
Acetylcholine
CHRM
Epinephrine, norepinephrine
ADR
Dopamine
DRD
Histamine
HRH
5-Hydroxytryptamine (5-HT)
HTR
Trace amine
TAR
Peptide
Angiotensin II, III
AGTR
Apelin
AGTRL1
Bombesin
GRPR
Bradykinin
BDKRB
Anaphylatoxin
C3a
C3AR
C5a
C5R
Lipoxin A4
FPRL
Cholecystokinin
CCKR
Endothelin
EDNR
Galanin
GALR
Ghrelin
GHSR
Kiss1 peptide
KISS1R
Melanocortin
α, β, γ-MSH
ACTH
MC1,3,4,5R
ACTH
MC2R
Motilin
MLNR
Neuromedin U
NMUR
Neuropeptide FF
NPFFR
Neuropeptide Y
NPYR
Neuropeptide W/B
GPR7/8
Neurotensin
NTSR
Opioids
OPR
Orexin
HCRTR
Oxytocin
OXTR
Somatostatin
SSTR
Tachykinin
Substance P
TACR1
Substance K
TACR2
Neuromedin K
TACR3
Urotensin II
UTS2R
Vasopressin
AVPR
Proteinase-activated like
PARRS
PAR
Prolactin-releasing peptide
PRLHR
Hormone protein
Melanin-concentrating hormone
MCHR
FSH
FSHR
LH
LHCGR
TSH
TSHR
Prostanoid
Prostaglandin PGD2
PTGDR
PGE2
PTGER
PGF2-alpha
PTGFR
PGI2
PTGIR
Thromboxane A2
TBXA2R
Nucleotide like
Adenosine
ADORA
Nucleotides
P2RY
Cannabinoid
Anandamide
CNR1
Platelet-activating factor
PTAFR
Gonadotropin-releasing hormone
GNRHR
Thyrotropin-releasing hormone
TRHR
Melatonin
MTNR
Lysophospholipid and LPA
Lysophosphatidic acid
EDGL
S1P, dihydro-S1P
EDGS
Leukotriene B4
LTB4R
Mas proto-oncogene
MAS1
Relaxin
RXFP
Cysteinyl-leukotriene
CYSLTR
Class B Secretin like
Calcitonin
CALCR
Corticotropin releasing hormone
CRHR
Gastric inhibitory peptide
GIPR
Glucagon
GCGR
Glucagon-like peptide
GLPR
Growth hormone-releasing hormone
GHRHR
Parathyroid hormone
PTHR
PACAP
PACAPR
Secretin
SCTR
Vasoactive intestinal peptide
VIPR
Class C Metabotropic glutamate / pheromone
Metabotropic glutamate
GRM
GABA
GABBR
Channels /other receptors
N-Acetylaspartyl-glutamate
GRIN
GABA
GABR
Acetylcholine
CHRN
Nucleotides
P2RX
Glutamate
L-Aspartate
L-Cysteic acid
L-Homocysteic acid
GRI
Glycine
β-Alanine
Taurine
GLR
N-Arachidonoyl-dopamine
N-Oleoyl-dopamine
Anandamide
Palmitoyl-ethanolamide
TRPV1
Porphyrins
BZRP
Cortisol
NR3C1
Growth hormone
GH
GHR
Triiodothyronine, thyroxine
THR
Leptin
LEP
LEPR
Prolactin
PRL
PRLR
Data on KEGG graph
Rendered by Pathview

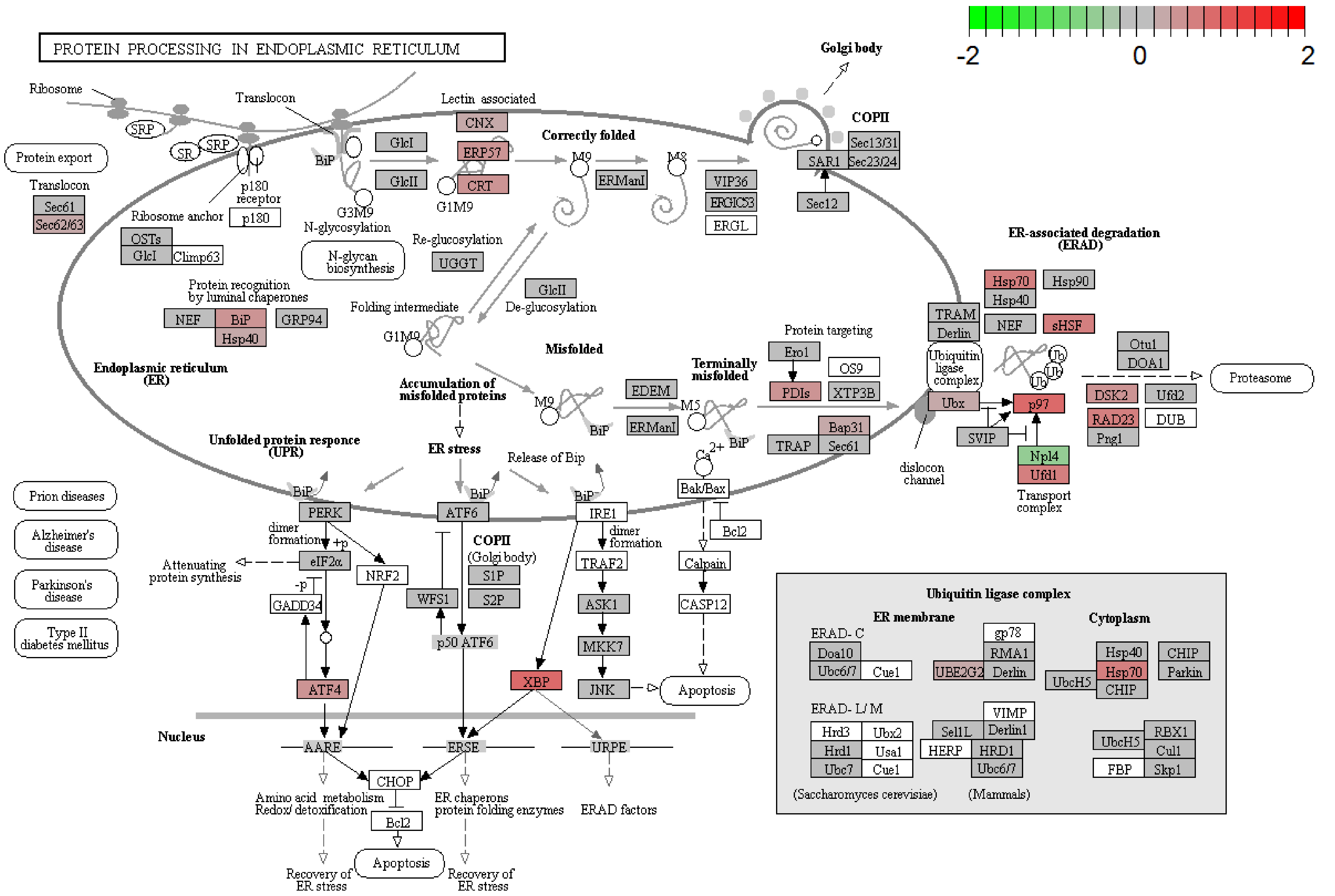

Data on KEGG graph
Rendered by Pathview
